# Supplementary material for: Experts’ views on translating NHS support to stop smoking in pregnancy into a comprehensive digital intervention
Source: PLOS Digit Health. 2024 Mar 27;3(3):e0000472. doi: 10.1371/journal.pdig.0000472 (PMC10971751; doi:10.1371/journal.pdig.0000472)
Supplement: S1 File — (DOCX) [file pdig.0000472.s001.docx]

**S1 File – Example Mentimeter activity**

**Figure A: Example Mentimeter polling results (session 1)**

**
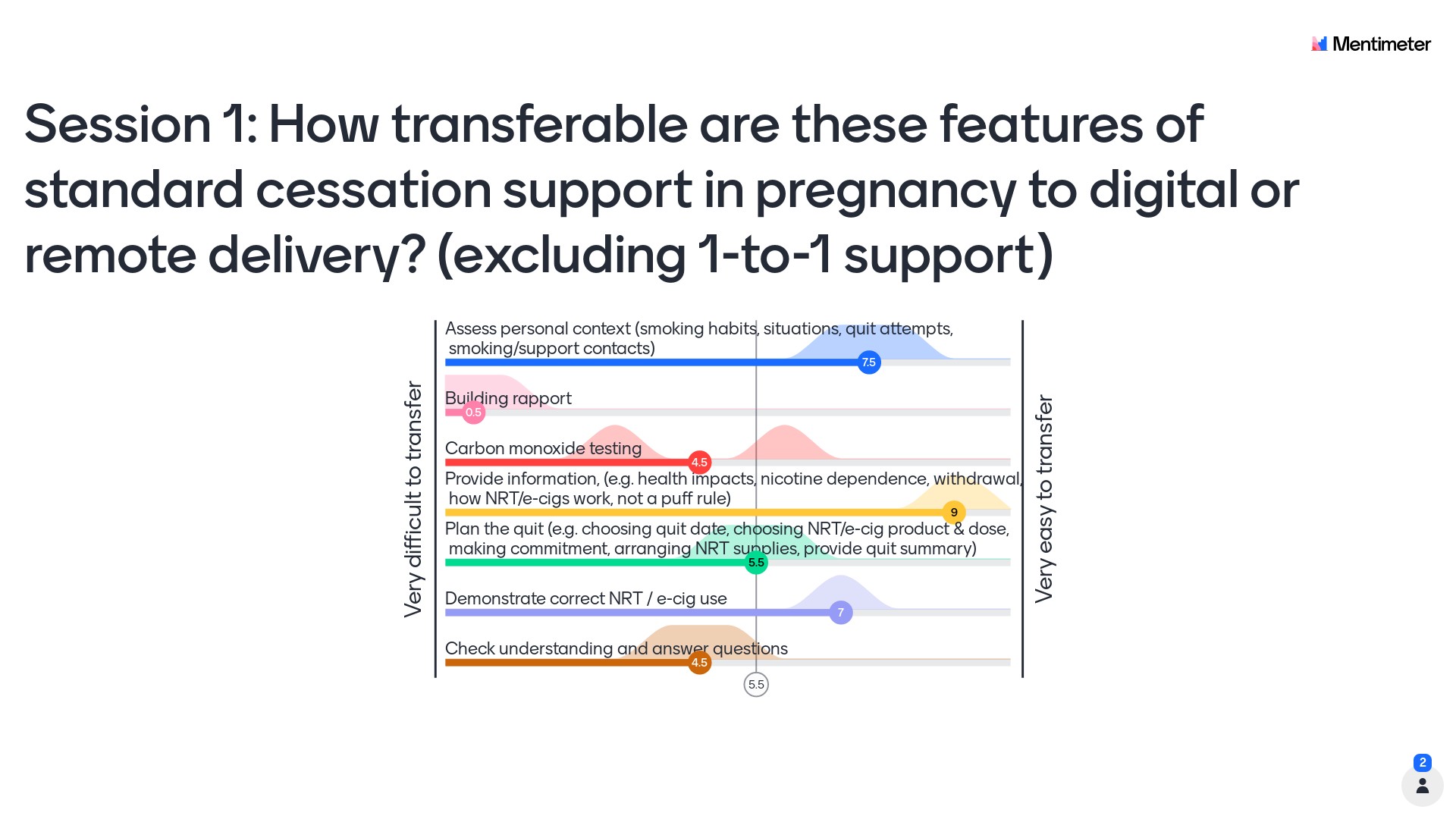
**

**Figure B: Example Mentimeter polling results (sessions 2-6)**

**
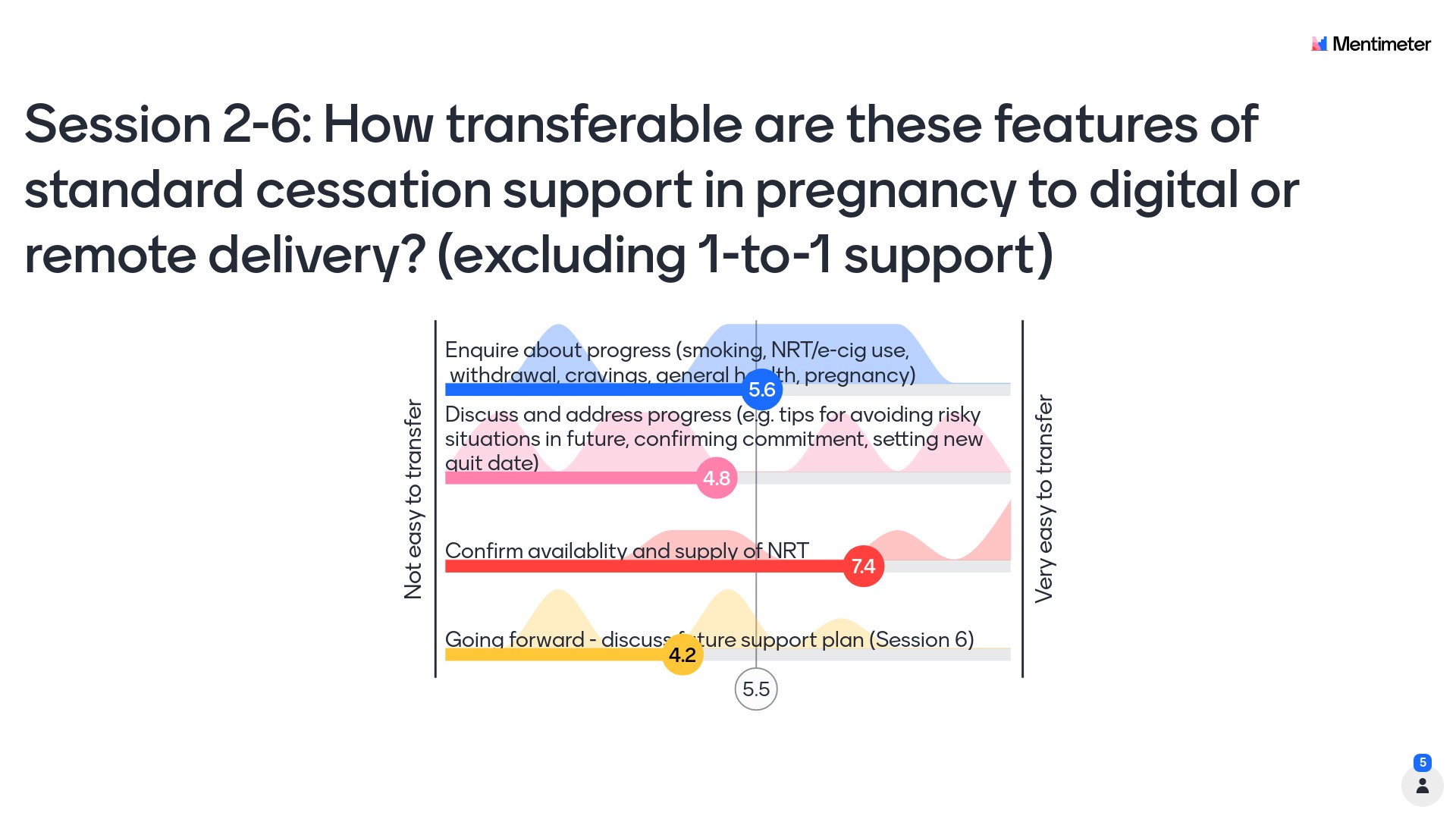
**
