# Supplementary material for: Experts’ views on translating NHS support to stop smoking in pregnancy into a comprehensive digital intervention
Source: PLOS Digit Health. 2024 Mar 27;3(3):e0000472. doi: 10.1371/journal.pdig.0000472 (PMC10971751; doi:10.1371/journal.pdig.0000472)
Supplement: S2 File — (DOCX) [file pdig.0000472.s002.docx]

**S2 File - Focus group topic guide for experts**

**(This guide will be adapted for one-to-one interviews)**

**Version 1.1 Date 14.03.22**

**Section A: 10 mins**

- Welcome, introduction of researchers and the eSupport to stop smoking in pregnancy study.
- The aim isn’t to challenge what is being done routinely but explore how routine support can be translated into digital support.
- Instructions regarding the focus group and ground rules.
- Explain the presence and purpose of recording.
- Address the issue of confidentiality.

**Introductions**

I’d like to find out some more about you by going round the group one at a time. Please tell me your first name and your experience in the field of smoking in pregnancy.

**Section B: 20 mins**

**1) Views about digital support**

- Ask participants to give examples of any digital tools for health that they have used or know about (e.g apps, websites, automated text messages) (capture these in a list)
  - Probe: any features they particularly liked about any health app, website etc. mentioned.
- Ask participants to give examples of any digital tools to help people stop smoking? Are participants aware of any digital tools specifically designed for stopping smoking in pregnancy?
  - Probe: any features they particularly liked about e-support tools mentioned.
- Open discussion on what they think about digital tools to help people stop smoking (Prompt: advantages, disadvantages, [for practitioners] scenarios where these have been discussed with pregnant people / who initiative this)

**Section C: 50 mins**

**2) Translating standard stop smoking support in pregnancy into digital**

The UK standard stop smoking treatment programme for pregnant people involves six sessions delivered by a trained advisor. Our idea is to move from this to digital support. Therefore, we would like your views on which parts of the standard treatment programme would be easier or more difficult to deliver this way.

- Identify general issues related to translating interpersonal counselling into digital support
- Identify specific issues that would need care and attention

**Session 1: Pre-quit assessment** *(polling activity)*

This session covers general preparations for quitting and it should aim to enhance motivation and boost self-confidence throughout.

- Assess personal context (smoking habits, situations, quit attempts, smoking/support contacts)
- Relationship and rapport
- Carbon monoxide testing
- Provide information (e.g. health impacts, nicotine dependence, withdrawal, how NRT/e-cigarettes work, not a puff rule)
- Plan the quit (e.g. choosing the quit date/ choosing NRT/ e-cigarette products & dose)
- Check understanding and answer questions
- Demonstrate correct NRT/ e-cig use

**Sessions 2 – 6 (combined)** *(polling activity )*

- Check progress
- Addressing progress
- Confirming availability and support of NRT
- Future support

Supplying NRT and CO monitoring are key parts of routine care, so now let’s explore potential remote ways of addressing these:

- Capture thoughts on offering NRT / CO monitoring to pregnant people alongside digitals support without interpersonal counselling (how might feedback elements be adapted to digital support)
- Identify examples of providing NRT / CO monitoring remotely during Covid – how was this done, client preferences / how could these be made as convenient as possible
  - Probe: If interviewees think that offering NRT without 'live' counselling will be an issue, could they suggest ways in which women could ask a question of a counsellor or flag a problem

**Section D: 20-30 mins**

**3) A future digital support package**

- **Generate a wish list of desired approaches and content for an digital support package to stop smoking in pregnancy (use visual prompts/ examples)**
  - [If not organically arisen] What are your thoughts on the different ways of delivering remote and digital support e.g. use of text messages, instant messaging (e.g. WhatsApp), a dedicated smartphone app, website, videos/audio etc.? Prompt – do you think it is better to have multiple channels or just one? Prompt – any key advantages/disadvantages with the different delivery modes mentioned? Prompt – could there be any groups that will likely struggle to access or engage with any of the above delivery modes?
- **Open discussion on how to engage pregnant people with digital tools to stop smoking in pregnancy**
  - Probe: those who don't manage to abstain completely, how we assess engagement e.g. task/question completion
- **Open discussion on how a digital support package might work alongside standard care**
- **Is there anything currently missing from standard care that digital support could provide?**
- **Prompt views on additional ideas [if time?]**
  - ‘Call centre’ for asynchronous support i.e. having someone always on hand to respond to message queries
  - Incentives for engaging in content/sessions
  - eSupport box sent in post (which would include CO monitor plus initial NRT and some other support tools/documents)
  - How best to link those engaging in digital support with interpersonal stop smoking service support?

**Section E: 10 mins**

**4) Close**

- A further phase of this study will interviews with pregnant people to seek their views. Are there particular questions you think we should be asking participants when we interview them?
- Thank the participants for sharing their views and ask them if there is anything else they would like to add. [Feel free to send on ideas]
