# Supplementary material for: Experts’ views on translating NHS support to stop smoking in pregnancy into a comprehensive digital intervention
Source: PLOS Digit Health. 2024 Mar 27;3(3):e0000472. doi: 10.1371/journal.pdig.0000472 (PMC10971751; doi:10.1371/journal.pdig.0000472)
Supplement: Attachment — Submitted filename: eSupport paper reviewer response 02.10.23.docx [file pdig.0000472.s003.docx]

School of Health Sciences

Edith Cavell Building

University of East Anglia

Norwich, NR4 7UL

Email: l.mcdaid@uea.ac.uk

2^nd^ Oct 2023

Dear Haleh Ayatollahi,

We are grateful to the reviewers for their helpful suggestions for improving our submitted manuscript. We have responded to comments and revised the manuscript, detailing our changes below, and hope you will now find it acceptable for publication. We thank you for your consideration and look forward to hearing from you.

Yours Sincerely,

Dr Lisa McDaid (on behalf of all authors).

Please note that page numbers given below related to the tracked change version of the manuscript.

Journal Requirements and editor comments:

**1. Please amend your detailed Financial Disclosure statement. This is published with the article. It must therefore be completed in full sentences and contain the exact wording you wish to be published.**

The following financial statement has now been added:

This article presents independent research funded by the National Institute for Health and Care Research (NIHR) Programme Development Grant NIHR203305 (awarded to FN). TC is an NIHR Senior Investigator. The views expressed are those of the author(s) and not necessarily those of the NIHR or the Department of Health and Social Care.

**2. We have noticed that you have uploaded Supporting Information files, but you have not included a list of legends. Please add a full list of legends for your Supporting Information files after the references list.**

A list of legends has now been added to the manuscript after the reference list. The supporting information has now been separated into two files linked to each legend.

S1. Example Mentimeter activity

S2. Focus group topic guide for experts

**3. Please add appropriate keywords using MeSH terms.**

Keywords are now MeSH terms.

Smoking cessation; pregnancy; cell phone; qualitative research; mobile applications

**4. Please follow the journal instructions for using appropriate headings, subheadings and organizing the manuscript.**

The manuscript has now been formatted in line with the PLos Digital Health guidance.

**5. The manuscript is a bit long. Please summarize the content or remove unnecessary paragraphs.**

The manuscript has been reviewed with unnecessary paragraphs removed. The previous submission main body was 8495 words. The revised submission is 7846, including additional text in response to reviewer comments.

**6. Reviewer #1: The only concern is if it would be actually implemented, would the patients lose personalized care to some extent?**

Thank you for this question. Our understanding of 'personalised care’ is when people have greater choice and control over the care they receive and have that care adapted to their characteristics and circumstances. If implemented, the digital stop smoking intervention for pregnancy would very likely be offered alongside interpersonal care, so that pregnant smokers could select the option (or combination of these) they prefer. Therefore, we would anticipate greater choice and personalisation of care.

**7. Reviewer #2: Page no-13 and may other areas: Author suggested e-cigarettes as a substitute of nicotine but not mentioned any health risk and safety issue of e-cigarettes during pregnancy as most e-cigarettes contain nicotine and other harmful substances besides nicotine. (1,2)**

References:
1. Quick facts on the risks of e-cigarettes for kids, teens, and young adults [Internet]. Centers for Disease Control and Prevention; 2022 [cited 2023 Aug 8]. Available from: https://www.cdc.gov/tobacco/basic_information/e-cigarettes/Quick-Facts-on-the-Risks-of-E-cigarettes-for-Kids-Teens-and-Young-Adults.html 
2. Association AL. Health risks of e-cigarettes and vaping [Internet]. [cited 2023 Aug 8]. Available from: <https://www.lung.org/quit-smoking/e-cigarettes-vaping/impact-of-e-cigarettes-on-lung>

While the long-term effects and any acute effects during pregnancy are unclear, there is emerging evidence which suggests that e-cigarettes appear to be more effective for cessation in pregnancy than nicotine patches (Hajek et al., 2022, Nature Medicine, doi: 10.1038/s41591-022-01808-0). In the UK, e-cigarettes are accepted as better than smoking in pregnancy and the standard treatment programme (STP) advises that if an expectant mother is interested in using an e-cigarette as part of a quit attempt, then stop smoking advisors should provide basic guidance on how to do this.

Standard Treatment Programme for Pregnant Women (p.28) <https://www.ncsct.co.uk/usr/pub/NCSCT%20Standard%20Treatment%20Programme%20for%20Pregnant%20Women.pdf>

We have now edited the background to highlight the uncertainty around e-cigarette safety in pregnancy (lines 112-118):

Similarly, while the safety of electronic cigarettes (e-cigarettes) in pregnancy remains unclear (32), there is a rapid emergence of evidence in this area, including a recent randomised control trial demonstrating their potential efficacy as a smoking cessation tool in pregnancy (33). E-cigarette use is supported in the STP (12) and therefore it is important to improve understanding of how e-cigarettes could potentially be provided remotely to increase cessation should provision become standard care in the future.

It is also noted in our findings section (lines 319-320) that experts from outside the UK that were interviewed had greater reservations about providing e-cigarettes as part of a digital cessation intervention in pregnancy.

**8. Most of the participants of the study argued about the future application of the proposed comprehensive digital intervention due to digital literacy, cost, technical issues, manipulation of the system by the users etc. So these should be considered and mentioned in the conclusion section.**

Thank you for drawing attention to these barriers to a comprehensive digital intervention for smoking cessation in pregnancy. They were alluded to in terms of ‘potential pitfalls’ but we have now edited the manuscript.

In the discussion section:

Likewise, the more complex the overall intervention becomes, and the more ‘active’ engagement from users, the greater the requirement is for digital literacy skills and competencies. (lines 516-518)

In the conclusion:

Likewise, CO monitoring and the supply of nicotine products could be delivered remotely but would require careful consideration of the potential pitfalls **such as technical issues and the fraudulent attempts to obtain products.** (lines 614-615)

**9. Involvement of Biomedical engineering experts to the procedure (both for study and review process) is recommended for identifying the health risk as well as technical issues**

Thank you for this recommendation. Any future intervention development work will involve an interdisciplinary team of academics and technical specialists.

**10. Reviewer #3: It is a hypothetical study to me with several non-corroborative subjective methods**

All qualitative research is intentionally subjective and fundamentally differs from quantitative methodologies in ontological and epistemological perspectives. Exploratory qualitative research, like used in our study, is an important step to lay the foundations for future quantitative and behavioural intervention development work by ensuring it is grounded in the perspectives of patients and professionals working in the field, and to help researchers and funders determine whether it is a worthy idea to pursue.

**11. How digital health can address this issue is largely unclear to me. Authors are requested to cite examples**

The reasons why many pregnant smokers do not make use of existing NHS interpersonal support are complex. A summary of evidence is provided in the background section, with some of these barriers being structural and others relating to pregnant smokers themselves. We have now expanded this section to highlight the potential advantages of digital approaches for overcoming barriers to accessing existing interpersonal models of smoking cessation treatment in pregnancy (lines 83-88):

Increasing the range of digital health tools available is a top priority for the NHS (21) and may help to circumvent some of the reasons why pregnant smokers do not use interpersonal cessation support; for example, digital support can improve convenience and be accessed at any time (22-24), it can increase anonymity and reduce the fear of feeling judged (23, 25), and it may be more attractive to underserved groups, such as young adults and teenagers (25).

**12. Duration of smoking and number of cigarettes smoked/day are two crucial criteria for assistive cessation of smoking habits. In the article, I haven't seen the mention of these while selecting the participants. Age group, comorbidities, and social determinants of health parameters are not included too, which I believe could influence the outcome very much.** 

Thank you for drawing attention to these important influences on tobacco use. For future testing of a comprehensive digital intervention for smoking cessation in pregnancy, we will collect baseline data in line with other randomised control trials we have undertaken in this field. This will include age; education, ethnicity, gestation; pre-pregnancy and current daily cigarettes smoked; nicotine dependence; strength and frequency of smoking urges; intention to quit; whether a quit date was set; partner's or significant other's smoking and health status. However, this information is not pertinent to this manuscript, where participants were selected primarily based on their professional experience and expertise.

**13. The neurobiology of smoking habit (although in a different perspective) can be cited "Chattopadhyay, S. (2022). Cigarette Smoking Erectile Dysfunction and its Extended Psychobiology. Adv Sex Reprod Health Res, 1(1), 124 -127."** 

We agree that the neurobiology of nicotine addiction has important implications for smoking cessation treatment; this is why we see nicotine substitution products augmenting the benefits of behavioural counselling. This study aimed to assess experts’ views on the digitalisation of an interpersonal treatment model for smoking cessation in pregnancy and we therefore do not feel that an explanation of neurobiology and mechanism is necessary to establish the context underlying this research study.

**14. How 'experts' are defined in this work is unclear. What are their experience levels in years? and most importantly, what are the anomalies and commonalities in their suggestions/advice/observations/recommendations?**

‘Expert’ was not defined by length of time in a role. We used purposive sampling to approach people with comprehensive knowledge in this field either known to the research team, working in prominent roles or who were specialist smoking cessation in pregnancy advisors/midwives. We have provided detail of the different expert categories in the manuscript, lines 159 – 164, which we have now edited for clarity.

Throughout the discussion section we have compared and contrasted perspectives. For example, in this paragraph below (lines 307 – 320) we highlighted how those working in a commissioning role had greater concerns about remote NRT provision and some international participants had safety concerns about the provision of NRT and e-cigarettes in pregnancy.

Most experts were unconcerned about providing NRT remotely without an advisor and believed it would enhance a digital cessation intervention […] However, some participants had reservations, particularly those involved in commissioning. One public health expert described it as a “*step too far*” (FG4-09-PTC), highlighting the potential governance and regulatory issues involved for those supplying the nicotine substitution products (“…*what happens if it then goes wrong and how to make sure that they're getting the right amount and the right product in the right way at the right time…”*-FG4-09-PTC). In addition, safety concerns were expressed by some participants from countries where NRT and e-cigarette use in pregnancy are not supported.

**15. Has any particular digital device been tested in this work? What is the outcome of the study?**

As discussed in the background section, a small number of digital smoking cessation interventions have already been tested in pregnancy with our team leading on a number of these. These have primarily focused on text message and internet delivery modes. We have now edited the section to highlight the particular interventions that have been developed and tested for pregnancy (lines 92-98):

However, few of these interventions are orientated towards pregnancy, which is the primary reason most pregnant smokers try to quit (28), and of those that are and have shown potential for cessation (29), **which are mainly computer- and text-based**, none have been routinely implemented in the NHS. There is also still much to learn about the best strategies to promote uptake and engagement with digital cessation interventions in this population, as this can impact on quitting success, and **low engagement is a key reason why a previous cessation app protype for pregnancy was abandoned** (30).
