# Supplementary material for: Experts’ views on translating NHS support to stop smoking in pregnancy into a comprehensive digital intervention
Source: PLOS Digit Health. 2024 Mar 27;3(3):e0000472. doi: 10.1371/journal.pdig.0000472 (PMC10971751; doi:10.1371/journal.pdig.0000472)
Supplement: Attachment — Submitted filename: Reviewer response 02.11.2023.docx [file pdig.0000472.s004.docx]

School of Health Sciences

Edith Cavell Building

University of East Anglia

Norwich, NR4 7UL

Email: l.mcdaid@uea.ac.uk

2^nd^ Oct 2023

Dear Haleh Ayatollahi,

We are thankful to you and the reviewers for considering our responses and revisions. We have now responded to the second round of comments and made further revisions to manuscript, detailing our changes below. We hope you will now find it acceptable for publication. We thank you for your consideration and look forward to hearing from you.

Yours Sincerely,

Dr Lisa McDaid (on behalf of all authors).

1. Please review your reference list to ensure that it is complete and correct. If you have cited papers that have been retracted, please include the rationale for doing so in the manuscript text, or remove these references and replace them with relevant current references. Any changes to the reference list should be mentioned in the rebuttal letter that accompanies your revised manuscript. If you need to cite a retracted article, indicate the article's retracted status in the References list and also include a citation and full reference for the retraction notice.

The reference list has been reviewed for completeness and checked for retractions. No retraction notices we identified. However, two incorrect DOI links have been amended (references 45 and 58).

2. Please amend your detailed Financial Disclosure statement. This is published with the article. It must therefore be completed in full sentences and contain the exact wording you wish to be published.

a. State what role the funders took in the study. If the funders had no role in your study, please state: “The funders had no role in study design, data collection and analysis, decision to publish, or preparation of the manuscript.”

b. If any authors received a salary from any of your funders, please state which authors and which funders.

The financial disclosure statement has been amended. The wording used for referencing the grant and TM as a senior NIHR investigator is stipulated by the NIHR.

‘This article presents independent research funded by the National Institute for Health and Care Research (NIHR) under the Programme Development Grant scheme (NIHR203305, awarded to FN, TC & JLB co-applicants, LM, PB, JE funded researchers). TC is an NIHR Senior Investigator. The views expressed are those of the authors and not necessarily those of the NIHR or the Department of Health and Social Care. The funders had no role in study design, data collection and analysis, decision to publish, or preparation of the manuscript.

Reviewer #2: A recent publication in the journal TOXICS has concluded that there is evidence to suggest that the use of e-cigarettes during pregnancy could potentially harm the health of both the mother and the fetus. The exposure to e-cigarettes is associated with various adverse effects such as increased systemic inflammation, low birth weight, preterm birth, and small size for gestational age status.
Ref: Vilcassim, M J Ruzmyn, et al. “Electronic Cigarette Use during Pregnancy: Is It Harmful?.” Toxics vol. 11,3 278. 18 Mar. 2023, doi:10.3390/toxics11030278
So, it is important to mention the harmful effects of electronic cigarettes (e-cigarettes) during pregnancy in conclusion for mass awareness and avoiding addiction to e-cigarettes instead of cigarette smoking.

We have now amended the conclusion to include a caution about the safety of e-cigarettes in pregnancy. However, we must emphasise that this research paper is not proposing that e-cigarettes are to be recommended in pregnancy, rather it is investigating views on providing them as part of a digital intervention should they become recommended as part of usual care in the UK.

‘Likewise, CO monitoring and the supply of ~~nicotine products~~ NRT and potentially e-cigarettes (if these become standard care in the future based on safety and effectiveness evidence) could be delivered remotely but would require careful consideration of the potential pitfalls, such as technical issues and the ability to misrepresent eligibility.’

Reviewer #3: While researching on pregnant smokers, I feel, understanding and giving a summary of neurobiology of smoking would help readers understanding the perspective in a better way. Researches have shown that pregnant women especially who are single, divorced, and separated (which are the SDoH parameters) smoke more. The key reason is to control their mood dysregulations during this phase of life. Therefore, I recommend authors to add a small section of 'Neurobiology of smoking'. I'm sure it'll strengthen the rationale of this work. Otherwise, I am happy with the explanations provided by the authors.

We stand by our previous rebuttal that we do not believe summarising the neurobiology of smoking is necessary for understanding the context of this paper, especially given its length. Smoking is a highly complex behaviour, and the standard treatment model uses evidence and behaviour change theory to address multiple modifiable influences (psychological, social, environmental and physical). In terms of SDoH parameters, the background section already notes that smoking in pregnancy is strongly associated with deprivation and part of the reason for undertaking this research is to help improve access to stop smoking support in pregnancy.

We were particularly interested in understanding if, and how, the more physiological aspect of nicotine dependence could be addressed in a digital intervention. We have now added a few words to emphasise the importance of this for helping to manage nicotine withdrawal.

‘To date, no digital cessation interventions for pregnancy have tried to replicate the combination of behavioural support and NRT, as used in the STP. The safe, remote, provision of NRT, which can help to address nicotine withdrawal and likely increases smoking cessation during pregnancy (31), could further enhance the effectiveness of digital support.’

While revising your submission, please upload your figure files to the Preflight Analysis and Conversion Engine (PACE) digital diagnostic tool, <https://eur01.safelinks.protection.outlook.com/?url=https%3A%2F%2Fpacev2.apexcovantage.com%2F&data=05%7C01%7Cl.mcdaid%40uea.ac.uk%7C02238a74196b4c65da8a08dbd3913f47%7Cc65f8795ba3d43518a070865e5d8f090%7C0%7C0%7C638336393004078907%7CUnknown%7CTWFpbGZsb3d8eyJWIjoiMC4wLjAwMDAiLCJQIjoiV2luMzIiLCJBTiI6Ik1haWwiLCJXVCI6Mn0%3D%7C3000%7C%7C%7C&sdata=tKvzfX521npP0UxMu1bcn8SPkdPuEq%2F2Yzty7SIg374%3D&reserved=0>. PACE helps ensure that figures meet PLOS requirements. To use PACE, you must first register as a user. Registration is free. Then, login and navigate to the UPLOAD tab, where you will find detailed instructions on how to use the tool. If you encounter any issues or have any questions when using PACE, please email PLOS at figures@plos.org. Please note that Supporting Information files do not need this step.

The two figures have now been uploaded and converted using the PACE digital diagnostic.

__________________________________________________
In compliance with data protection regulations, you may request that we remove your personal registration details at any time.  (Use the following URL: <https://eur01.safelinks.protection.outlook.com/?url=https%3A%2F%2Fwww.editorialmanager.com%2Fpdig%2Flogin.asp%3Fa%3Dr&data=05%7C01%7Cl.mcdaid%40uea.ac.uk%7C02238a74196b4c65da8a08dbd3913f47%7Cc65f8795ba3d43518a070865e5d8f090%7C0%7C0%7C638336393004078907%7CUnknown%7CTWFpbGZsb3d8eyJWIjoiMC4wLjAwMDAiLCJQIjoiV2luMzIiLCJBTiI6Ik1haWwiLCJXVCI6Mn0%3D%7C3000%7C%7C%7C&sdata=weMtrvfB1Hp6wuwMBHhWw4sxej91fsA7TqlUklIE7tA%3D&reserved=0)>. Please contact the publication office if you have any questions.
