# Supplementary material for: Experts’ views on translating NHS support to stop smoking in pregnancy into a comprehensive digital intervention
Source: PLOS Digit Health. 2024 Mar 27;3(3):e0000472. doi: 10.1371/journal.pdig.0000472 (PMC10971751; doi:10.1371/journal.pdig.0000472)
Supplement: Attachment — Submitted filename: Reviewer response 08.01.2024.docx [file pdig.0000472.s005.docx]

School of Health Sciences

Edith Cavell Building

University of East Anglia

Norwich, NR4 7UL

Email: l.mcdaid@uea.ac.uk

8^th^ Jan 2024

Dear Haleh Ayatollahi,

We are thankful to you and the reviewers for considering our responses and revisions. We have now responded to the third round of comments. We hope you will now find it acceptable for publication. We thank you for your consideration and look forward to hearing from you.

Yours Sincerely,

Dr Lisa McDaid (on behalf of all authors).

Reviewer #3: Please bring the Social Determinants of Health (SDoH) parameters connected to neurobiology behind smoking in pregnancy and let the readers know how the issue could be managed by digitized interventions that the article is proposing. If not considered, I believe, any intervention will be highly inadequate and the end goal can not be achieved. Thank you.

We stand by our previous two rebuttals on this point unless the Editor wishes for the change to be made. We are not seeking to develop a ‘new’ intervention, we are exploring perceptions on whether the standard treatment programme for smoking in pregnancy, which is routinely delivered in the UK but has low uptake, can be delivered digitally rather than by stop smoking advisors - and to establish what the barriers and facilitators to this might be. Addressing social context was one of the identified barriers reported in the findings (highlighted in the revised version). We have now been awarded funding to carrying out further research to co-design the digital intervention and will need to consider any limitations identified in this study.

Some social and cultural influences are mediated by modifiable determinants, e.g. outcome expectancies, which can be addressed, and partner support could influence the social-environment – both are in the standard treatment programme and can be incorporated into the digital intervention. Ensuring the digital intervention is relevant and adapted to under-served populations by tailoring to specific barriers to quitting faced by pregnant smokers from these groups will be essential. Social deprivation is a key determinant of smoking in pregnancy. Therefore, by making pregnancy cessation support more accessible to a broader range of people, we will hopefully begin to address some health inequalities in maternal smoking.
