# Supplementary material for: Experts’ views on translating NHS support to stop smoking in pregnancy into a comprehensive digital intervention
Source: PLOS Digit Health. 2024 Mar 27;3(3):e0000472. doi: 10.1371/journal.pdig.0000472 (PMC10971751; doi:10.1371/journal.pdig.0000472)
Supplement: Attachment — Submitted filename: Reviewer response 31.01.2024.docx [file pdig.0000472.s006.docx]

School of Health Sciences

Edith Cavell Building

University of East Anglia

Norwich, NR4 7UL

Email: l.mcdaid@uea.ac.uk

31^st^ Jan 2024

Dear Haleh Ayatollahi,

We have now responded to the reviewer comment on social determinates of health and made further revisions to manuscript, detailing our changes below. We hope you will now find it acceptable for publication. We thank you for your consideration and look forward to hearing from you.

Yours Sincerely,

Dr Lisa McDaid (on behalf of all authors).

1) Reviewer #3: Please bring the Social Determinants of Health (SDoH) parameters connected to neurobiology behind smoking in pregnancy and let the readers know how the issue could be managed by digitized interventions that the article is proposing. If not considered, I believe, any intervention will be highly inadequate and the end goal can not be achieved. Thank you.

(Dear authors, I'm still sticking to my point as a reviewer. It has not been addressed in the revised manuscript. To me, it is therefore inadequate for publication. However, I leave it to the editor of PLOS Digital Health to take the decision)

We have added a sentence and reference to the background section outlining the determinates of smoking behaviour in pregnancy.

Smoking in pregnancy is associated with numerous pregnancy complications and life-long conditions for babies (1-3). While maternal smoking rates have declined in high-income countries (4), there are socio-demographic differences in who is more likely to smoke when becoming pregnant and who is more likely to quit. Deprivation is overwhelmingly associated with both, reinforcing health inequalities (5, 6). Along with living in more unfavourable financial and social conditions, psychological factors, dependence on tobacco and access to effective and acceptable support, can all interact to make quitting smoking in pregnancy more difficult (7).

7. Ingall G, Cropley M. Exploring the barriers of quitting smoking during pregnancy: A systematic review of qualitative studies. Women Birth. 2010;23(2):45-52. doi: <https://doi.org/10.1016/j.wombi.2009.09.004>

We have also provided more detail about the content of the standard treatment programme for smoking cessation in pregnancy and that this is designed to be an individual-level intervention.

These guidelines are implemented through the ‘Standard Treatment Programme’ (STP) (13), an evidence-based intervention designed to be delivered by specialist stop smoking advisors. It consists of 1) a pre-quit assessment 2) setting a quit date, 3) weekly behavioural counselling sessions, 4) regular carbon monoxide (CO) monitoring, and 5) the provision of free NRT with encouragement to use two forms (patch plus fast-acting product). The weekly sessions are designed to address modifiable determinates of smoking at an individual level, such as enhancing motivation to quit, planning how deal with tempting situations and cravings, and addressing any NRT usage issues. Offering support to family and friends alongside the STP is also recommended.

Finally, we have added a new paragraph and additional reference to the limitations section. This outlines the limitations of a behavioural treatment intervention, especially in terms of addressing wider social determinates of smoking. However, it also lists some potential strategies that could be incorporated into the digital intervention to help respond to social context.

Additionally, consideration must be given to the limitations of digitalising the STP for smoking in pregnancy. Addressing the social determinants of smoking requires a broader range of actions than an individual-level intervention can address alone. Additional population-level interventions will therefore continue to be needed (62). However, a digital intervention offers the potential to enable greater equity in access to stop smoking services in pregnancy. Strategies can also be embedded within the digital intervention to help respond to social context such as tailoring to individual characteristics, providing coping strategies, offering support for family and friends, and signposting to other support services.

62. Boucher J, Konkle AT. Understanding inequalities of maternal smoking - bridging the gap with adapted intervention strategies. IJERPH. 2016;13(3):282.

2) Please amend your detailed Financial Disclosure statement. This is published with the article. It must therefore be completed in full sentences and contain the exact wording you wish to be published.

a. State the initials, alongside each funding source, of each author to receive each grant.
b. State what role the funders took in the study. If the funders had no role in your study, please state: “The funders had no role in study design, data collection and analysis, decision to publish, or preparation of the manuscript.”
c. If any authors received a salary from any of your funders, please state which authors and which funders.

If you did not receive any funding for this study, please simply state: “The authors received no specific funding for this work.”

Previous statement

This article presents independent research funded by the National Institute for Health and Care Research (NIHR) under the Programme Development Grant scheme (NIHR203305, awarded to FN, TC & JLB co-applicants, LM, PB & JE funded researchers). TC is an NIHR Senior Investigator. The views expressed are those of the authors and not necessarily those of the NIHR or the Department of Health and Social Care. The funders had no role in study design, data collection and analysis, decision to publish, or preparation of the manuscript.

Revised statement

This article presents independent research funded by the National Institute for Health and Care Research (NIHR) under the Programme Development Grant scheme (NIHR203305). The funding was awarded to FN (Principal Investigator), TC & JLB (co-applicants), and LM, PB & JE received all or part of their salary from the funder. TC is an NIHR Senior Investigator. The views expressed are those of the authors and not necessarily those of the NIHR or the Department of Health and Social Care. The funders had no role in study design, data collection and analysis, decision to publish, or preparation of the manuscript.
